# Comparative Genomic Analyses Reveal Potential Factors Responsible for the ST6 Oxacillin-Resistant *Staphylococcus lugdunensis* Endemic in a Hospital

**DOI:** 10.3389/fmicb.2021.765437

**Published:** 2021-11-25

**Authors:** Shih-Cheng Chang, Lee-Chung Lin, Jang-Jih Lu

**Affiliations:** ^1^Department of Laboratory Medicine, Linkou Chang Gung Memorial Hospital, Taoyuan, Taiwan; ^2^Department of Medical Biotechnology and Laboratory Science, College of Medicine, Chang Gung University, Taoyuan, Taiwan; ^3^Department of Medicine, College of Medicine, Chang Gung University, Taoyuan, Taiwan

**Keywords:** oxacillin-resistant *Staphylococcus lugdunensis*, mobile genetic elements, prophage, multidrug-resistant genes, resistant islands

## Abstract

Oxacillin-resistant *Staphylococcus lugdunensis* (ORSL) is considered a life-threatening isolate in healthcare settings. Among ORSL clones, ST6-SCC*mec* II strains are associated with an endemic spread in hospitals. We analyzed the complete genome of ORSL CGMH-SL118, a representative strain. Results revealed that this strain contained three MGEs (two prophages and one plasmid) other than the SCC*mec* II element, which showed remarkable differences in genome organization compared to the reference strains from NCBI. Eight multidrug-resistant genes were identified. All but *blaZ* were carried by MGEs, such as the SCC*mec* II element [*mecA*, *ant* (9)-Ia, and *ermA*] and the prophage φSPbeta [*aac* (6')-*aph* (2'), *aph* (3')-III, and *ant* (6)-Ia], indicating that MGEs carrying multidrug-resistant genes may be important for ST6 strains. The prophage φSPbeta contains *sasX* gene, which was responsible for the pathogenesis of *Staphylococcus aureus*. A phage-mediated resistant island containing *fusB* (SlRI_*fusB*-118_) was found near φSPbeta, which was highly homologous to type III SeRI_*fusB*-5907_ of *Staphylococcus epidermidis*. In contrast to previous studies, over 20% of ST6 isolates showed a fusidic acid-resistant phenotype, suggesting that phage-mediated intraspecies transmission of resistant islands may become an important issue for ST6 strains. Sixty-eight clinical isolates of ST6 *Staphylococcus lugdunensis* (50 OSSL, oxacillin-sensitive *S. lugdunensis*, and 18 ORSL, including CGMH-SL118) collected from various types of specimens in the hospital were studied. Among these isolates in this study, ORSL showed similar drug-resistant genes and phenotypes as CGMH-SL118. The comparative genomic analyses highlight the contribution of MGEs in the development and dissemination of antimicrobial resistance in ST6 strains, suggesting that resistance determinants and virulence factors encoded by MGEs provide a survival advantage for successful colonization and spread in healthcare settings.

## Introduction

*Staphylococcus lugdunensis* was considered a commensal coagulase-negative staphylococcal species (CoNS) until the emergence of nosocomial infections, after which it became an important pathogen (Yeh et al., 2015). Unlike other CoNS, *S. lugdunensis* showed similar pathogenicity to *Staphylococcus aureus* (Argemi et al., 2015; Douiri et al., 2016; Heilbronner and Foster, 2021) and caused various infections, such as skin and soft tissue infections, bone and joint infections, bacteraemia, and infective endocarditis (Wu et al., 2011; Yeh et al., 2015, 2016; Douiri et al., 2016). The mortality rate of endocarditis caused by *S. lugdunensis* infection is nearly 40% (Liu et al., 2010), while that caused by other CoNS is only 14.3% (Fernandez-Rufete et al., 2012; Molina et al., 2013), suggesting that *S. lugdunensis* is more virulent than other CoNS. Previous epidemiological surveillance showed that most isolates remained susceptible to oxacillin (Lin et al., 2015; Yeh et al., 2015, 2016); however, a significantly high proportion of oxacillin-resistant *S. lugdunensis* (ORSL) strains were found in nosocomial infections (Lin et al., 2015), indicating that transfer of SCC*mec* (staphylococcal cassette chromosome *mec*) should be monitored in hospitals. In fact, our previous investigation identified an endemic spreading in northern Taiwan caused by a group of SCC*mec* II, ST6 strains (Cheng et al., 2015). Further characterization of their SCC*mec* cassette structure revealed similarities with *S. aureus*, which suggested that ORSL may act as an interspecies for SCC*mec* transfer to *S. aureus* in hospitals (Chang et al., 2019).

It is interesting to note that the endemic spread was caused by SCC*mec* II, ST6 strains, since most ORSL isolates belong to SCC*mec* V strains (Yeh et al., 2015, 2016). According to our previous studies regarding ORSL antimicrobial susceptibilities, SCC*mec* II strains showed more multidrug-resistant phenotypes than SCC*mec* V strains (Yeh et al., 2015), suggesting that drug resistance may enhance SCC*mec* II endemic spread. In addition to the drug resistance, virulence factors may also enhance the endemic spread. Although few studies have revealed virulence factors associated with their pathogenicity (Giormezis et al., 2015; Argemi et al., 2017b; Heilbronner and Foster, 2021), the above evidence originated from OSSL, while studies involving ORSL infections are still limited.

Whole-genome sequencing analysis can provide more comprehensive information to investigate virulence factors, drug resistance, pathogenesis, and other factors, which have been widely used in *S. aureus* studies (Kuroda et al., 2001; Laabei et al., 2014). A prospective study of these reports found that most virulence factors were encoded by mobile genetic elements (MGEs), such as plasmids, prophages, or pathogenicity islands (Malachowa and DeLeo, 2010). *Staphylococcus lugdunensis* N920143 was one of the earlier strains that provided complete genome sequence and information about virulence factors, although few MGEs were found in this strain compared to *S. aureus* (Heilbronner et al., 2011). Recent comparative genomic analyses have identified several novel MGEs in *S. lugdunensis*, and few of the pathogenicity islands have been reported thus far (Argemi et al., 2017a, 2018; Lebeurre et al., 2019).

Recently, the first complete genome sequence of *S. lugdunensis* ORSL JICS135 revealed the presence of several virulence features compared to other OSSLs (Shibuya et al., 2020). The SCC*mec* element of JICS135 contained two genes encoding microbial surface components recognizing adhesive matrix molecules (MSCRAMM)-like proteins, which are considered responsible for strain pathogenesis (Patti et al., 1994). However, structural comparisons indicated that partial regions of SCC*mec*_JICS135_ were similar to those of two previously reported ORSL SCC*mec* V strains: CMUH 22 and CMUH25 (Chang et al., 2017). To elucidate the undiscovered virulence factors in SCC*mec* II, ST6 ORSL, whole-genome sequence analysis was adopted to decipher its genome structure. The present study demonstrated that SCC*mec* II and ST6 strains contained unique MGEs encoding a putative virulence factor and antimicrobial resistance genes, which may be responsible for its endemic spread.

## Materials and Methods

### Bacterial Isolates

All *S. lugdunensis* isolates were collected from 2009 to 2014 at Taiwan Linkou Chang Gung Memorial Hospital. Strain CGMH-SL118 was isolated from a blood sample and selected for whole-genome sequencing analysis. Sixty-eight clinical isolates of ST6 *S. lugdunensis* (50 OSSL, oxacillin-sensitive *S. lugdunensis*, and 18 ORSL, oxacillin-resistant *S. lugdunensis*, including CGMH-SL118) collected from various types of specimens in the hospital were studied, which had been published (Yeh et al., 2015, 2016); detailed information is included in Supplementary Table 1.

### Whole-Genome Sequencing and Annotation

**Figure 1:**
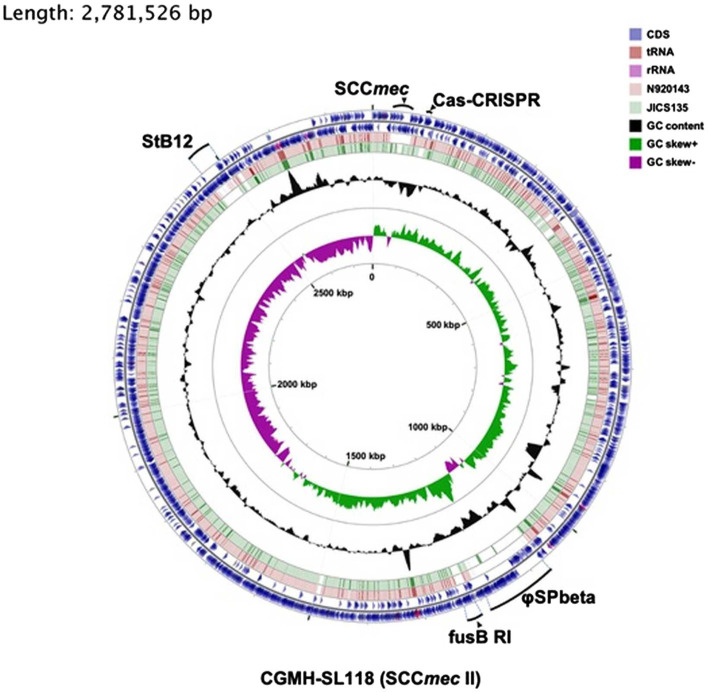
Comparative genomic analysis of three *Staphylococcus lugdunensis* strains. Six rings for the circular diagram (inner to outer): GC skew of the CGMH-SL118, GC content of the CGMH-SL118, green ring representing the genome of JICS135, pink ring representing the genome of N920143, and two of the outer blue rings representing two coding strands (forward and reverse) of CGMH-SL118. On these two outer blue circles, three colors represent different gene types: blue represents coding sequence, shallow pink represents tRNA, and deep pink represent rRNA. mobile genetic elements (MGEs; prophage; StB12 and phi-SPbeta, SCC*mec*, *fusB*-resistant island) and CRISPR-Cas regions were labeled with bold dark.

*Staphylococcus lugdunensis* strain CGMH-SL118 was grown on TSB medium overnight for genomic DNA extraction. The extracted genomic DNA was sequenced using the PacBio™ method (Pacific Biosciences, Menlo Park, CA, United States). A single total length 2,818,231 base pair contig was generated using three software; a *de novo* assembler Flye (Kolmogorov et al., 2019) was used for contig assembly, contigs scaffolding was applied using SSPACE (Boetzer and Pirovano, 2014), and scaffolds were finally polished using arrow algorithm.^1^ Gene annotation was generated using “Prokka v1.12”,^2^ which is designed for bacterial or viral genome annotation. The quality of the assembled genome was evaluated using “Quast v4.5” (Gurevich et al., 2013). The annotated data were further verified using the RAST web annotation service^3^ to determine the function of each gene. CGview web service^4^ was used for visualization of the circular genome and comparative genomic analysis of three individual strains: CGMH-SL118, N920143 (Heilbronner et al., 2011), and JICS135 (Shibuya et al., 2020; Figure 1). The prophage search was performed using “PHAST” (PHAge Search Tool)^5^ and “PHASTER” analyses.^6^ The Cas-Crispr system was verified using the CRISPR web server.^7^ Virulence factors were identified using the “Virulence Factors of Pathogenic Bacteria Database” (VFDB)^8^ web service, and antimicrobial resistance genes were analyzed using “The Comprehensive Antibiotic Resistance Database” (CARD).^9^

### Antimicrobial Phenotype and Relative Drug-Resistant Genes Characterization

The antimicrobial phenotype was characterized using the disk diffusion method. Gentamicin, clindamycin, erythromycin, and fusidic acid disks were placed on the surface of the bacterium-grown medium, and their susceptibilities were evaluated *via* the inhibition zone. All procedures and evaluations were performed according to the CLSI guideline (Wayne, 2018). The prevalence of drug-resistant genes among the collected isolates was examined using PCR. Primers used in this study are listed in Table 1. PCR conditions for the detection of drug-resistant genes [including *aac* (6′)-*aph* (2′), *aph* (3′)-III, *ant* (6)-Ia, *ant* (9)-Ia, *fusB*, and *ermA*] were performed as previously described (Sutcliffe et al., 1996; Kao et al., 2000; Sepulveda et al., 2007; Castanheira et al., 2010; Fessler et al., 2010; Emaneini et al., 2013).

**Table 1:** PCR primer sets used in this study.

Target	Primer	Sequence (5'–3')	Size	References
*SasX*	SasX-F	GCACATGCAGCTGATTATGTAAATG	463	This study
	SasX-R	CTAAACCAGAATTAGATTGTCCGCC		
*muts/sasX* ^*^	Prophage_L-F	TCTAGGCGCTCCTTATTCGT	5,752	This study
	Prophage_L-R	TGCTCCCGCTAATGTAGTTGT		
*Hyp/yeeE3’* ^*^	Prophage_R-F	TTTGAGATACTGTTTTATTCGCTTT	2,203	This study
	Prophage_R-R	TGATCGTCCAGTAATGCAAAA		
*aac-aph*	aac-aph-F	GAGCAATAAGGGCATACCAAAAATC	505	Kao et al., 2000
	aac-aph-R	CCGTGCATTTGTCTTAAAAAACTGG		
*aph-IIIa*	aph (3')-IIIa-F	GGCTAAAATGAGAATATCACCGG	526	Emaneini et al., 2013
	aph (3')-IIIa-R	CTTTAAAAAATCATACAGCTCGCG		
*ant-Ia*	ant (6')-Ia-F	CCTTATTGCCCTTGGAAGAGT	580	Sepulveda et al., 2007
	ant (6')-Ia-R	TCAGCGGCATATGTGCTATC		
*fusB*	FusB-F	TCATATAGATGACGATATTG	439	Castanheira et al., 2010
	FusB-R	ACAATGAATGCTATCTCGAC		
*ermA*	ermA-F	TCTAAAAAGCATGTAAAAGAA	645	Sutcliffe et al., 1996
	ermA-R	CTTCGATAGTTTATTAATATTAGT		
*spc (ant(9)-Ia)*	spc-F (ant (9)-Ia)	ACCAAATCAAGCGATTCAAA	561	Fessler et al., 2010
	spc-R	GTCACTGTTTGCCACATTCG		

* Targeting the left (between *muts* and *sasX* genes) and right (between hypothetical protein and *yeeE3’* genes) junctions of the prophage φSPbeta.

## Results

### Information of CGMH-SL118 Whole-Genome Sequence

**Figure 2:**
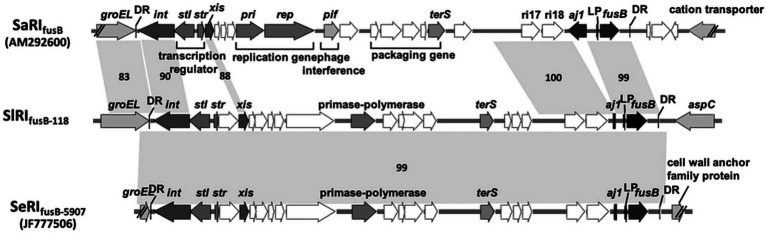
Comparison of *fusB*-resistant islands between CGMH-SL118 and the other species. Three of the *fusB* resistance island from *Staphylococcus lugdunensis* CGMH-SL118 (SlRI_fusb-118_), *Staphylococcus epidermidis* JF777506 (SeRI_fusb-5907_), and *Staphylococcus aureus* AM292600 (SlRI_fusb-118_) were comparatively analyzed the structure similarities. The gray color and double slash were representing the inserted location on each genome; the right side of these three islands was inserted below the *groEL*: the left side was varied by each strain. The dark color represents integrase (*int*), excisionase (*xis*), *fusB* core region genes (*aj1* and *fusB*), and two sequences (LP; *aj1*-leader peptide, and DR; direct repeat sequence). The other genes were labeled with white color. Critical genes are additionally labeled with the abbreviation; *stl* and *str* are transcription regulators; *pri* and *rep* are responsible for replication; *pif* is response for phage interference; *terS* is phage terminase small subunit, which is responsible for phage packaging. The number accompanied with shadow connected to each *fusB* resistance island represents the sequence similarities.

To understand the difference in genome structure composition with that of *S. lugdunensis*, the whole-genome sequence of CGMH-SL118 was compared with those of *S. lugdunensis* strains N920143 and JICS135 (Table 2 and Figure 1). The genome size of CGMH-SL118 was 2,818,231bp, which is larger than that of the other two strains (JICS135, 2,687,768bp and N920143, 2,595,888bp, Table 2). The CGMH-SL118 genome also contained more coding sequences (2,659 encoded proteins) than the other two strains (N920143, 2,406 and JICS135, 2,498, Table 2). The overall genome coverage of CGMH-SL118 was 93 and 97% for JICS135 and N920143 strains, respectively. Both coverage regions showed >99% identity, indicating that most genome regions were conserved among the three strains. The total numbers of tmRNA, tRNA, and rRNA, and GC content percentages were identical between CGMH-SL118 and JICS135, which were also similar to N920143 (Table 2). In addition to these conserved regions, structural genome differences can still be found among these three strains, and most belonged to MGEs (Figure 1). Both the CGMH-SL118 and JICS135 strains were ORSL with the SCC*mec* element, but N920143 was OSSL without the SCC*mec* element (Table 2). Among these three strains, only CGMH-SL118 contained a plasmid, which was similar to the plasmid SAP107A in *Staphylococcus epidermidis*. In addition to the SCC*mec* element and plasmid, PHASTER analysis showed that both CGMH-SL118 and N920143 contained prophages, with CGMH-SL118 carrying φSPbeta and StB12 and N920143 carrying φSL1 (Heilbronner et al., 2011). Figure 1 shows the genomic sequence comparison of CGMH-SL118 with JICS135 and N920143. Two spaces on JICS135 and N920143 represented the prophage regions (StB12 and φSPbeta-like), which only existed on CGMH-SL118. A region near the prophage φSPbeta, which appeared as a blank space in the corresponding regions of N920143 and JICS135, was further investigated. This region contained a novel fusidic acid-resistant island (named SlRI_*fusB*-118_) of *S. lugdunensis* (*fusB* RI, Figure 1), which was almost identical to type III SeRI_*fusB*-5907_ (JF777506) of *S. epidermidis*, but partially similar to type I SaRI (AM292600) of *S. aureus* (Chen et al., 2011; Figure 2). Sequence blast results showed that all three fusidic acid-resistant islands were inserted near the *groEL* gene. The SaRI was only partially aligned with SlRI_*fusB*-118_ in two regions: the left insertion region (83–90% identity) and *fusB* encoding region (>99% identity). In contrast, SeRI_*fusB*-5907_ showed high similarity with SlRI_*fusB*-118_ across all islands.

**Table 2:** Basic information of CGMH-SL118 whole-genome sequence analysis and comparison with other reference strains.

	CGMH-SL118	JICS135	N920143
Size (bp)	2,818,231	2,687,768	2,595,888
Number of CDS	2,659	2,498	2,406
Clinical origin	Blood	Blood	Breast abscess
tmRNA	1	1	1
tRNA	61	61	55
rRNA	19	19	16
G+C content	33.7%	33.7%	33.8%
Plasmid	1 (36,705bp)	0	0
Prophage	2	0	1
SCC*mec* (bp)	39,029	92,958	–
Crispr	1	0	1
**Genome fraction vs. CGMH-SL118**			
Query cover	–	93%	97%
Identity	–	99.57%	99.96%

### Prevalence of Antibiotic-Resistant Genes Between CGMH-SL118 and Other Reference Strains

**Figure 3:**
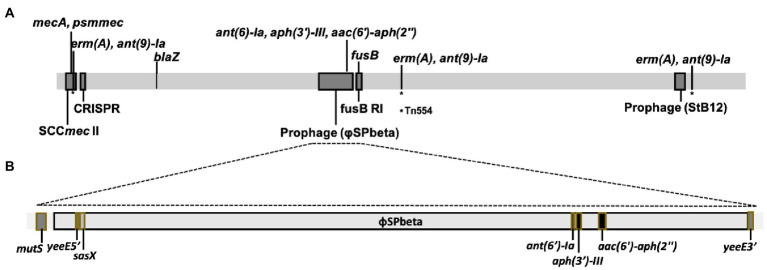
Schematic presentation of genome structure of CGMH-SL118. **(A)** The linear schematic presentation of the CGMH-SL118 genome; which containing all of the antibiotic resistance genes, MGEs (SCC*mec* and prophage), and CRISPR. Detailed structure of prophage Spbeta-like was shown in **(B)**, containing each antibiotic resistance genes and a putative virulence gene. This prophage was inserted in the *yeeE* (nearby the *mutS*), which caused the *yeeE* separated into two parts on this structure. The asterisk (*) represents the position of Tn554.

An antibiotic resistance gene survey identified several drug resistance genes involved in various resistance mechanisms in CGMH-SL118, which differed from strains N920143 and JICS135 (Table 3). A previous study has shown that OSSL N920143 has no antibiotic resistance gene (Heilbronner et al., 2011). In contrast, ORSL JICS135 has only three antibiotic resistance genes [*blaZ, mecA*, and *aac* (6')-*aph* (2'); Shibuya et al., 2020], all of which were also identified in CGMH-SL118. Interestingly, except for *mecA* carried on the SCC*mec* element, the other two genes were located in the genomic regions of CGMH-SL118, which differed from JICS135. In CGMH-SL118, *blaZ* was located close to the SCC*mec* element, and *aac* (6')-*aph* (2') was found in the prophage φSPbeta region (Figure 3A). In addition to these three genes, other antibiotic resistance genes were also identified in CGMH-SL118, including three aminoglycoside [*aph* (3′)-III, *ant* (6)-Ia, *ant* (9)-Ia], one fusidic acid (*fusB*), and one macrolide (*ermA*)-resistant genes. Among these drug-resistant genes, three aminoglycoside-resistant genes were located on the prophage φSPbeta-like, which was located close to SlRI_*fusB*-118_. CGMH-SL118 harbored multiple copies of *ermA* and *ant* (9)-Ia; one was located on the SCC*mec* cassette, and the other two were located aside to two prophage regions. Moreover, we observed *ant* (9)-Ia co-localized with *ermA* on the transposon Tn*554* (Figure 3A).

**Table 3:** Comparison of antibiotic-resistant gene distribution between CGMH-SL118 and other reference strains.

Gene name	Strain			Product	Function	Antibiotics
	CGMH-SL118	JICS135	N920143			
*blaZ*	Chromosome	Chromosome	–	Beta-lactamase	Beta-lactam resistance	Penicillin, oxacillin
*mecA*	Chromosome (SCC*mec*)	Chromosome (SCC*mec*)	–	Penicillin-binding protein 2a	Beta-lactam resistance	Amoxicillin, cefepime cefoxitin
*aac (6')-aph (2")*	Chromosome (prophage)	Chromosome	–	Aminoglycoside modifying enzyme	Aminoglycoside resistance	Gentamicin, streptomycin, kanamycin
*aph (3')-III*	Chromosome (prophage)	–	–	Aminoglycoside modifying enzyme		
*ant (6)-Ia*	Chromosome (prophage)	–	–	Ant (6)-Ia protein		
*ant (9)-Ia*	Chromosome Chromosome (SCC*mec*)	–	–	Streptomycin 3"-adenylyltransferase		
		–	–			
*fusB*	Chromosome (Phage-mediated resistant island)	–	–	Fusidic acid resistance protein	Fusidic acid resistance	Fusidic acid
*erm (A)*	Chromosome Chromosome (SCC*mec*)	–	–	Erythromycin resistance protein	Macrolide, Lincosamide, and Streptogramin B resistance	Erythromycin
		–	–			

### Surveillance of Antibiotic-Resistant Phenotypes Among ST6 Clinical Isolates

The presence of multidrug-resistant genes in CGMH-SL118 made us consider whether this phenotype is a general distribution scheme among other clinical isolates of ST6 *S. lugdunensis*. Sixty-eight clinical isolates of ST6 were screened for the presence of aminoglycoside, macrolide, and fusidic acid resistance genes (Table 4). All 18 ORSL isolates carried SCC*mec* II. The prevalence of four aminoglycoside-resistant genes was over 90% among ORSL clinical isolates, and the prevalence was consistent with the results of the antibiotic susceptibility test, indicating that nearly 90% of ORSL isolates were resistant to gentamycin (Table 4). Similar trends were also observed for the macrolide-resistant phenotype. All 18 ORSL isolates with the *ermA* gene were resistant to clindamycin and erythromycin. However, only five isolates harbored *the fusB* gene, and four also showed a fusidic acid-resistant phenotype.

**Table 4:** Prevalence of antibiotic resistance genes and phenotype among clinically collected ST6 strains.

Antibiotics	Resistance gene	No. (%) of isolates with the specific antimicrobial resistance genes and phenotypes			
		ORSL (*n*=18)		OSSL (*n*=50)	
		Resistance phenotype	Resistance gene	Resistance phenotype	Resistance gene
Gentamicin	*ant(6')-Ia*	16 (88.9)	17 (94.4)	4 (8)	1 (2)
	*aph(3')-III*		17 (94.4)		8 (16)
	*aac(6')-aph(2")*		18 (100)		18 (36)
	*ant(9)-Ia*		17 (94.4)		1 (2)
Fusidic acid	*fusB*	4 (22.2)^*^	5 (27.8)	15 (30)^*^	16 (32)
Clindamycin	*ermA*	18 (100)	18 (100)	16 (32)	1 (2)
Erythromycin	*ermA*	18 (100)	18 (100)	15 (30)	1 (2)

* No significant difference between oxacillin-resistant *Staphylococcus lugdunensis* (ORSL) and OSSL (*p*=0.53).

Previous studies have shown that the ST6-SCC*mec* II strains are the major persistent clones in hospitals (Cheng et al., 2015). Therefore, we were also interested in the antibiotic susceptibilities of ST6 OSSL isolates (Table 4). Compared with the ORSL, most antibiotic resistance genes were not present in ST6 OSSL isolates, which was consistent with their antibiotic-resistant phenotypes. The *fusB* was the only exception; both the antibiotic-resistant phenotype and prevalence of *fusB* were higher than ORSL, and statistical analysis showed no significant difference between them.

### Prevalence of Prophage φSPbeta and Its Encoded Virulent and Antibiotic-Resistant Genes Among ST6 Clinical Isolates

The presence of prophage was the major difference between CGMH-SL118 and JICS135. Although two prophage regions were identified in CGMH-SL118, only the φSPbeta region contained antibiotic-resistant genes (Figure 3B). Three aminoglycoside resistance genes [*aph* (3′)-III, *ant* (6)-Ia, and *aac (6′)-aph (2′)*] within the prophage φSPbeta indicated its contribution to the drug-resistant phenotype. A previous study has shown that this prophage carries the *sasX* gene, which is an important virulence factor for *S. aureus* pathogenesis (Li et al., 2012). To understand the prevalence of this prophage among ST6 OSSL and ORSL clinical isolates, gene-specific PCRs were used to examine the existence of φSPbeta and its encoding virulence factor *sasX* and three antibiotic resistance genes (Table 5). A high prevalence of φSPbeta was found in ST6 ORSL (16/18), and these 16 isolates also had *sasX* and three antibiotic-resistant genes. Only two ST6 ORSL isolates did not have this prophage, which is quite different from the distribution in ST6 OSSL isolates. Although some of the above genes could still be found in isolates lacking this prophage, most of these four genes were found simultaneously with the φSPbeta in the isolates.

**Table 5:** Distribution of prophage and its relative genes in ST6 *Staphylococcus lugdunensis* clinical isolates.

ST6	No. (%)	Prophage	*sasX*	*ant (6')-Ia*	*aph (3')-III*	*aac (6') aph (2")*
ORSL (*n*=18)	16 (88.9)	+	+	+	+	+
	1 (5.6)	−	+	+	+	+
	1 (5.6)	−	−	−	−	+
OSSL (*n*=50)	1 (2)	+	+	+	+	+
	1 (2)	−	−	−	+	+
	6 (12)	−	−	−	+	−
	16 (32)	−	−	−	−	+
	26 (52)	−	−	−	−	−

## Discussion

Epidemiological surveillance showed that most clinical isolates of *S. lugdunensi*s belonged to OSSL, and only a few isolates belonged to ORSL (McHardy et al., 2017). Among the ORSL clinical isolates, SCC*mec* V strains were the most frequently isolated strains, yet our previous studies showed that some isolates belonging to SCC*mec* II were the strains possibly involved in the endemic transmission, which makes them important in the hospital environment (Cheng et al., 2015; Yeh et al., 2015). To further understand the factors contributing to transmission of SCC*mec* II strains, one of these isolates, CGMH-SL118, was selected for whole-genome sequencing and compared with other OSSLs and SCC*mec* V ORSL deposited in the NCBI database. The complete genome sequence of *S. lugdunensi*s CGMH-SL118 showed a different genome composition compared to that of OSSL or SCC*mec* V ORSL. Comparative genome analysis showed that CGMH-SL118 contained two prophages, which may cause an increase in genome size (Table 2). In addition to the prophage, only CGMH-SL118 contained one plasmid, and the above information indicated that MGEs played key roles in differences observed in SCC*mec* II ORSL compared to other ORSLs and OSSLs.

Mobile genetic elements are known for their horizontal gene transfer abilities and are involved in the dissemination of drug-resistant genes and virulence factors among various strains. Our results showed that multiple drug-resistant genes were present in the CGMH-SL118 genome, and most were located inside the MGEs. The SCC*mec* II element of CGMH-SL118 contained not only the *mecA* gene, but also two additional drug-resistant genes, *ermA* and *ant (9)-Ia*. Since these two genes confer erythromycin and clindamycin resistance, SCC*mec* II strains are probably more difficult to treat than SCC*mec* V strains. According to epidemiological surveillance, few resistant isolates of the above three antibiotics have been reported in previous studies (Taha et al., 2019). Therefore, these two resistant genes were not commonly found in *S. lugdunensis*. Among ST6 clinical isolates, almost all ORSLs showed resistance to erythromycin and clindamycin, but only a few OSSLs showed similar phenotypes (Table 4), indicating that erythromycin and clindamycin resistances were highly correlated with the SCC*mec* cassette.

Most of these antibiotic-resistant genes were located on MGEs, which suggested that antibiotic resistance genes horizontally transferred between bacterial strain and indeed species. This possibility is supported by our previous studies showing that the SCC*mec* cassette of CGMH-SL118 was structurally similar to that of *S. aureus* N315, and both belonged to SCC*mec* II (Chang et al., 2019), but structurally different from SCC*mec* V carried by JICS135 (SCC*mec*_MRSL-JICS135_) (Shibuya et al., 2020). In this study, a transposon Tn*554* containing *ermA* and adjacent *ant*(9)-Ia was identified in the SCC*mec* II element of CGMH-SL118 (Chang et al., 2019), and three copies of Tn*554* were found in the CGMH-SL118 genome. This phenomenon was also found in the *S. aureu*s N315 genome, suggesting that multiple copies of Tn*554* were commonly observed in SCC*mec* II containing staphylococci.

In addition to the SCC*mec* cassette, our genome sequencing data revealed the presence of two prophage regions, which was the major difference between SCC*mec* II and SCC*mec* V ORSL. The φSPbeta region contained three antibiotic resistance genes and a putative virulence factor *sasX* gene, all of which might able to enhance the competitiveness of this strain in the healthcare environment. *Ant (6′)-Ia*, *aph (3′)-III*, and *aac (6′)-aph (2′)* all belong to the aminoglycoside-resistant genes, which also contributed to the gentamicin-resistant phenotype. The varying prevalence of these three genes among ST6 ORSL and OSSL indicated that their distribution was highly correlated with the MGEs, since all three genes were located in the φSPbeta region (Table 3). This result suggesting that the dissemination of drug-resistant determinants may carried out by the prophage φSPbeta through a horizontal transfer process.

In addition to aminoglycoside resistance genes, a putative virulence factor SasX reportedly plays critical roles in strain pathogenesis (Li et al., 2012). The SasX found in *S. aureus* is a homolog of SesI (*S. epidermidis* surface protein) and contains the LPXTG motif, which can be recognized by sortase A (SrtA) and anchored on the cell wall (Soderquist et al., 2009). Virulence studies of *S. epidermidis* showed that the *sesI* gene was only present in clinical isolates but not in the flora of healthy individuals, which indicates a virulence factor associated with strain pathogenesis (Soderquist et al., 2009). Further epidemiological surveillance of *S. aureus* showed that *sasX* was transferred by prophage φSPbeta from *S. epidermidis* and broadly existed in ST239 *S. aureus*, which was continuously transferred into ST59 *S. aureus* and considered to be a critical factor for increased clonal spreading (Li et al., 2012). In coagulase-negative staphylococci, *sasX* has been observed mainly in *S. haemolyticus* and *S. epidermidis* isolates, but not in *S. lugdunensis* isolates (Tekeli et al., 2020). To the best of our knowledge, this is the first report of *sasX* found in *S. lugdunensis*. Moreover, further epidemiological surveillance of ST6 clinical isolates showed that both *sasX* and prophage φSPbeta were present in most ORSLs, but only in one OSSL clinical isolate, suggesting that this putative virulence factor may enhance the ST6 ORSL during the endemic infection.

Among the various drug resistances identified in *S. lugdunensis*, fusidic acid resistance is one of the phenotypes that should be noted. Surveillance of CoNS found that less than 10% of CoNS showed fusidic acid-resistant phenotypes (Farrell et al., 2016). Similar results have been reported in previous studies of *S. lugdunensis* and indicated that fusidic acid resistance was low in *S. lugdunensis* (Hellbacher et al., 2006; Zaaroura et al., 2018). The whole-genome sequence revealed that CGMH-SL118 contained the *fusB* gene, which is responsible for the fusidic acid-resistant phenotype. Further investigation showed that both OSSL and ORSL ST6 isolates were more than 20% resistant (Table 4), suggesting that fusidic acid resistance is becoming a threatening issue in *S. lugdunensis*. Previous studies of fusidic acid resistance in *S. epidermidis* showed that *fusB*-mediated fusidic acid resistance was carried by various types of phage-related resistant islands (Chen et al., 2011, 2013), which were responsible for the high fusidic acid resistance phenotype in *S. epidermidis* (Chen et al., 2011). It has been suggested that *fusB* RI may be easily transferred and spread among different species (Chen et al., 2011). The structure of SlRI_fusB-118_ was almost identical to that of SeRI_fusB-5907_ and contained partial portions of SaRI, suggesting that the *fusB*-resistant island may be transferred between two CoNS species and further transferred to *S. aureus*. Although this phenomenon was rarely mentioned in *S. lugdunensis* (Bocher et al., 2009; Taha et al., 2019), it appears consistent with our previous studies that CGMH-SL118 may act as a reservoir for interspecies transfer of MGEs, such as SCC*mec* elements, to *S. aureus* in hospitals. This drug resistance gene was only found in SCC*mec* II ORSL in our analysis, suggesting that the antibiotic resistance properties of SCC*mec* II strains may differ from those of other SCC*mec* type ORSLs. In addition, our ST6 clinical isolates showed an increased fusidic acid-resistant phenotype, which may be considered as an alternative factor causing ST6 endemic transmission in hospital environments.

This study investigated the whole-genome sequence of SCC*mec* II, ST6 ORSL, and identified several MGEs containing multidrug-resistant genes and virulence factor, which could account for the differences observed when compared to SCC*mec* V ORSL and OSSL. The unique genome composition of CGMH-SL118 suggests that it may take advantage of these multidrug-resistant genes and virulence factor to compete with other OSSLs and ORSLs in hospital environments. After further surveillance of clinical isolates, we found that drug-resistant phenotypes similar to that of CGMH-SL118 were commonly found among ST6 ORSLs. Taken together, our results demonstrate that MGEs containing multidrug-resistant genes and virulence factor may play critical roles in ST6-SCC*mec* II ORSL infections and may be one of the reasons for endemic transmission in hospitals.

## Data Availability Statement

Raw data of the CGMH-SL118 complete genome sequence were submitted to the NCBI BioProject and approved with accession number CP048008.

## Ethics Statement

This study was approved by the Ethics Committee of Linkou Chang Gung Memorial Hospital. Because this study only experimented on bacteria and did not affect the patients adversely, the Review Board agreed the usage of the requested bacterial materials from Linkou Chang Gung Memorial Hospital bacterial storage.

## Author Contributions

S-CC, L-CL, and J-JL conceived the study, analyzed the data, prepared the tables and figures, and contributed to the writing of the manuscript. All authors read and agreed to the published version of the manuscript.

## Funding

This work was supported by grants from the Ministry of Science and Technology, Taiwan (MOST 110-2320-B-182A-006-MY3, 108-2320-B-182A-013, and 110-2811-B-182A-505).

## Conflict of Interest

The authors declare that the research was conducted in the absence of any commercial or financial relationships that could be construed as a potential conflict of interest.

## Publisher’s Note

All claims expressed in this article are solely those of the authors and do not necessarily represent those of their affiliated organizations, or those of the publisher, the editors and the reviewers. Any product that may be evaluated in this article, or claim that may be made by its manufacturer, is not guaranteed or endorsed by the publisher.

## Footnotes

^1^ https://github.com/PacificBiosciences/GenomicConsensus

^2^ https://github.com/tseemann/prokka/

^3^ https://rast.nmpdr.org/

^4^ http://cgview.ca/

^5^ http://phast.wishartlab.com/index.html

^6^ http://phaster.ca/

^7^ https://crispr.i2bc.paris-saclay.fr/

^8^ http://www.mgc.ac.cn/VFs/main.htm

^9^ https://card.mcmaster.ca/
