# Supplementary material for: Comparative Genomic Analyses Reveal Potential Factors Responsible for the ST6 Oxacillin-Resistant Staphylococcus lugdunensis Endemic in a Hospital
Source: Front Microbiol. 2021 Nov 25;12:765437. doi: 10.3389/fmicb.2021.765437 (PMC8655729; doi:10.3389/fmicb.2021.765437)
Supplement: Supplementary file 1 [file Data_Sheet_1.PDF]

Supplementary Table 1. Clinical information of 68 ST6 strains used in this analysis

No.	Specimen Type*	SCC*mec*	Oxacillin	*sasX*	Drug resistant gene					
					ant(6)	*aph(3')-3*	*aac-aph*	*fusB*	*ermA*	*ant(9)-la*
1	B	II	R	+	+	+	+	-	+	+
2	AM	-	S	-	-	-	-	-	-	-
3	B	II	R	+	+	+	+	-	+	+
4	B	II	R	+	+	+	+	-	+	-
5	B	-	S	-	-	-	-	-	-	-
6	B	-	S	-	-	-	-	-	-	-
7	B	-	S	-	-	-	+	+	-	-
8	B	-	S	-	-	-	-	+	-	-
9	B	II	R	+	+	+	+	-	+	+
10	B	II	R	+	+	+	+	-	+	+
11	SY	-	S	-	-	-	+	-	-	-
12	B	-	S	-	-	-	+	-	+	+
13	B	-	S	-	-	-	+	-	-	-
14	B	II	R	+	+	+	+	-	+	+
15	B	-	S	-	-	-	-	-	-	-
16	B	-	S	-	-	-	-	-	-	-
17	B	II	R	+	+	+	+	-	+	+
18	B	-	S	-	-	-	+	-	-	-
19	B	II	R	+	+	+	+	+	+	+
20	B	-	S	-	-	-	-	-	-	-
21	B	-	S	-	-	-	+	+	-	-
22	BF	-	S	-	-	-	-	-	-	-
23	B	-	S	+	+	+	+	+	-	-
24	B	-	S	-	-	-	-	+	-	-
25	B	-	S	-	-	-	-	-	-	-
26	B	-	S	-	-	-	-	+	-	-
27	B	II	R	+	+	+	+	+	+	+
28	AS	-	S	-	-	-	-	+	-	-
29	B	-	S	-	-	-	+	+	-	-
30	B	-	S	-	-	-	-	+	-	-
31	B	II	R	+	+	+	+	-	+	+
32	B	II	R	-	-	-	+	-	+	+
33	B	II	R	+	+	+	+	+	+	+
34	B	-	S	-	-	-	-	+	-	-
35	B	II	R	+	+	+	+	-	+	+

36	B	-	S	-	-	-	-	-	-	-
37	B	II	R	+	+	+	+	+	+	+
38	B	II	R	+	+	+	+	+	+	+
39	DTS	-	S	-	-	-	+	-	-	-
40	DTS	-	S	-	-	-	-	+	-	-
41	DTS	-	S	-	-	-	-	-	-	-
42	DTS	-	S	-	-	-	-	-	-	-
43	DTS	-	S	-	-	-	-	-	-	-
44	DTS	-	S	-	-	-	-	-	-	-
45	DTS	-	S	-	-	-	+	-	-	-
46	PUS	-	S	-	-	-	+	-	-	-
47	WD	-	S	-	-	-	-	-	-	-
48	WD	-	S	-	-	-	-	-	-	-
49	WD	II	R	+	+	+	+	-	+	+
50	PUS	-	S	-	-	-	-	+	-	-
51	WD	-	S	-	-	-	+	-	-	-
52	AB	-	S	-	-	-	+	-	-	-
53	OTH	-	S	-	-	-	-	-	-	-
54	OTH	-	S	-	-	-	+	-	-	-
55	PUS	-	S	-	-	-	+	-	-	-
56	PUS	II	R	+	+	+	+	-	+	+
57	PUS	-	S	-	-	+	-	+	-	-
58	WD	-	S	-	-	-	-	-	-	-
59	PUS	-	S	-	-	-	-	-	-	-
60	OTH	-	S	-	-	-	+	+	-	-
61	PUS	-	S	-	-	+	-	-	-	-
62	PUS	-	S	-	-	+	+	-	-	-
63	WD	-	S	-	-	-	+	-	-	-
64	PUS	-	S	-	-	+	-	-	-	-
65	WD	-	S	-	-	+	-	-	-	-
66	AB	-	S	-	-	+	-	+	-	-
67	PUS	-	S	-	-	+	-	+	-	-
68	B	untyped	R	+	+	+	+	-	+	+

* Specimen type abbreviations: AB: abscess; AM: amniotic fluid; AS: ascites; B: blood; BF: body fluid; DTS: deep tissue; OTH: other; PUS: pus; SY: synovial fluid; WD: wound.
